# *phnE* frameshift reversion frequency: beyond density effects to genomic prevalence

**DOI:** 10.1128/jb.00073-26

**Published:** 2026-07-14

**Authors:** Luísa Andrea Villanueva da Fonseca, Marina Caldas Leite, Beny Spira

**Affiliations:** 1 Departamento de Microbiologia, Instituto de Ciências Biomédicas Universidade de São Paulo 28133, São Paulo, Brazil; University of Virginia School of Medicine, Charlottesville, Virginia, USA

**Keywords:** *phnE*, reversion, mutant frequency, allele

## Abstract

Phosphonates are alternative phosphorus sources widely present in environmental settings and in commercially relevant compounds such as pesticides and antibiotics. In *Escherichia coli*, uptake and assimilation of phosphonates are mediated by the 14-gene *phnCDEFGHIJKLMNOP* operon. In the laboratory, strain K-12 and its derivatives, *phnE*, encoding the phosphonate permease, carry an 8 bp insertion that causes a frameshift and premature termination. Early studies reported unusually high frequencies of *phn* revertants selected on phosphonates as the sole phosphorus source. Separately, high cell density has been proposed to suppress mutation emergence in multiple systems. Here, we reexamined the frequency of *phn* revertants in *E. coli* K-12. When phosphate impurities were rigorously eliminated from the medium, estimated revertant frequencies decreased by orders of magnitude, contradicting earlier reports. Revertant frequency was not significantly affected by bacterial density on selective plates. Furthermore, under phosphate-limiting conditions, the presence of a functional *phnE* allele did not confer a measurable fitness advantage. Finally, an *in silico* survey revealed that approximately 15% of *E. coli* strains lack functional *phn* genes, suggesting that loss of phosphonate utilization may be favored in specific ecological or physiological contexts.

**IMPORTANCE:**

Phosphonates are an important phosphorus reservoir in many environments, and their utilization by *Escherichia coli* depends on the *phn* operon. This study reexamines the unusually high frequencies of *phnE* revertants reported for laboratory K-12 strains. By controlling for phosphate contamination in selective media, we show that reversion frequencies are substantially lower than previously estimated and largely independent of cell density. We further find that restoration of *phnE* function does not confer a detectable fitness advantage under phosphate limitation. Consistent with this observation, genomic analyses reveal that loss of *phn* function is common among natural *E. coli* isolates. Together, these findings suggest that loss of *phn* function may represent an adaptive strategy in a substantial fraction of natural populations.

## INTRODUCTION

Bacteria can assimilate phosphorus from various sources, including inorganic phosphate (Pi), phosphate esters, organophosphates, and phosphonates (Pn). Pi is the preferred phosphorus source and, together with phosphonates and certain phosphate esters, forms the group of compounds that are directly transported into the cell without prior modification (1, 2). Phosphonates are characterized by a direct carbon-phosphorus covalent bond, such as 2-aminoethylphosphonate, methylphosphonate, and phosphonate esters. They account for about 10% of the phosphorus available in the oceans (3, 4). Phosphonates also have clinical and commercial relevance: fosfomycin is a naturally produced antibiotic from *Streptomyces*, glyphosate—one of the most widely used agricultural herbicides—is a synthetic organophosphonate, and bisphosphonates, widely used for the treatment and prevention of osteoporosis, reduce fracture risk and bone resorption (5).

When the concentration of Pi in the medium becomes limiting, many microorganisms shift to phosphonates as an alternative phosphorus source. In *Escherichia coli*, the ability to metabolize Pn relies on the *phn* operon, a 10.9 kb locus composed of 14 genes, whose expression is regulated by Pi availability. These genes (*phnCDEFGHIJKLMNOP* or *phn* operon for short) encode proteins involved in phosphonate transport and catabolism (4). The *phn* operon belongs to the PHO regulon, a set of more than 30 genes mostly involved in Pi acquisition that respond to Pi shortage. When the concentration of Pi in the medium drops to <4 M, the histidine kinase PhoR autophosphorylates and transfers the Pi moiety to PhoB, the response regulator. Once phosphorylated, PhoB dimerizes and binds to the PHO boxes (a DNA consensus sequence that replaces the −35 promoter sequence in all PHO regulatory regions, including *phnC*), and interacts with the RNA polymerase associated with σ^70^, initiating the transcription of all PHO genes and operons.

The *phnCDE* genes encode an ABC-type membrane transporter responsible for importing phosphonates, phosphate, and phosphite (6, 7). Within this complex, *phnC* encodes the ATP-binding subunit, *phnD* the periplasmic substrate-binding protein, and *phnE* the transmembrane domains. Through comparative analysis of *phn* operon sequences from *E. coli* K-12 and B strains, reference 8 showed that the inability of K-12 strains to metabolize phosphonates is due to a frameshift in *phnE* caused by the insertion of an 8 bp octamer in a repetitive 5′-ABB-3′ sequence (5′-CGCTGGCG TGCTGGCG TGCTGGCG), whereas B strains carry only the 5′-AB-3′ repeat. The authors also showed that revertant mutants derived from K-12 capable of using phosphonate had lost this extra octamer, restoring the reading frame and re-establishing phosphonate utilization. Reported reversion frequencies of the *phn^−^* genotype in K-12 strains selected on medium containing phosphonate as the sole phosphorus source vary considerably, ranging from >10^−2^ to 2 × 10^−5^ (9, 10). It has been proposed that the instability in *phnE* arises from a strand-slippage mispairing mechanism during replication, facilitated by the presence of the 5′-CTG-3′ trinucleotide, the binding site for the DnaG primase (9).

Some studies have reported that cellular density might influence the frequency of mutants in bacteria (11, 12) across several mutational systems. Krasovec et al. (11) reported a negative association between mutation rate and cell density in *E. coli* such that lowering population density can increase the mutation rate at least threefold, while Neves et al. (12) showed a 100-fold reduction in the frequency of PHO-constitutive mutants in *E. coli* in high-cell-density (10^9^ cells) plates.

In the present study, we revisited the frequency of reversion of the 8 bp octamer insertion in *phnE* that renders phosphonate transport non-functional in the *phn^−^* K-12 strain MG1655. We show that growth of wild-type cells on the selective plates likely influenced earlier estimates of *phn^−^* revertant frequency (RF), whereas bacterial density does not appear to affect the frequency of *phn^+^* revertants. In addition, the fitness of the *phn^−^* mutant was examined, along with the prevalence of this mutation among clinical and environmental *E. coli* isolates.

## RESULTS

### Effect of bacterial density on the frequency of *phn^+^* revertants

Given the reported influence of bacterial density on various mutation systems (11, 12), we sought to determine whether cell density also affects the RF of the 8 bp insertion in *E. coli* K-12 *phnE*. Previous studies have reported *phn^+^* revertant frequencies ranging from a remarkably high 3.4−8.6 × 10^−2^ (9) to 2 × 10^−5^ (10). However, those studies did not consider the possibility that the phosphonate source—methylphosphonate—was contaminated with inorganic phosphate (Pi). Even trace amounts of Pi could support bacterial growth for several generations and artificially inflate the apparent frequency of revertants, as shown by the well-documented effect of carbon source contaminants on the selection of *lac* mutants (13–16). We therefore quantified the Pi content of the components of TGPn medium—a minimal medium containing aminoethylphosphonic acid (AmPn) as the sole phosphorus source, which is routinely used as the selective medium to isolate *phn^+^* revertants. Trace amounts of Pi were detected in AmPn, corresponding to a final concentration of approximately 15 μM in TGPn selective plates (each plate contains 1 mM AmPn). No detectable Pi was found in the other medium components (T-salts, glucose, or agar).

**Fig 1:**
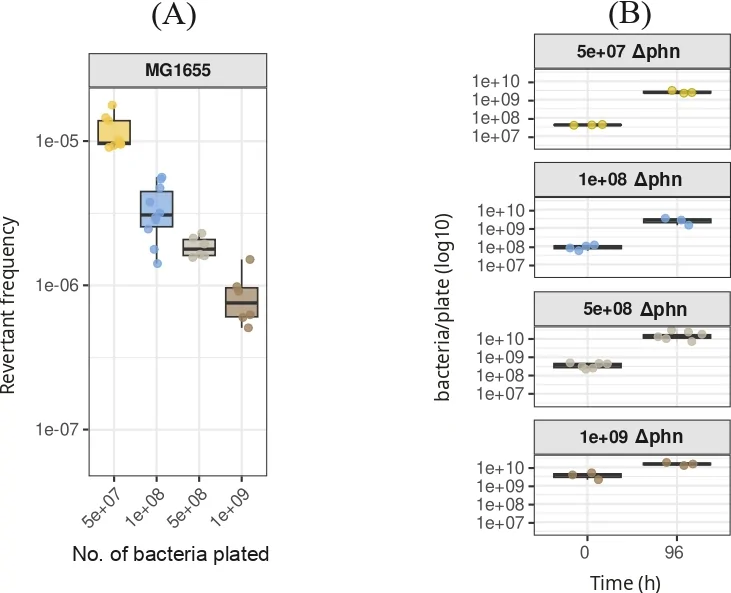
Frequency of *phn^+^* revertants and growth of Δ*phnCDE* on TGPn plates. (**A**) Approximately 5 × 10^7^, 10^8^, 5 × 10^8^, and 10^9^ MG1655 cells were plated on TGPn plates followed by incubation at 37°C for 4 days. To obtain the RF, the number of *phn^+^* colonies on each plate was divided by the actual initial number of cells obtained by CFU counting on L-agar plates. ANOVA followed by Games-Howell *post hoc* comparisons showed that the 5 × 10^7^ group is statistically different from the others with *P*-values <0.001. The *P*-values of the comparisons 10^8^ vs 5 × 10^8^, 10^8^ vs 10^9^, and 5 × 10^8^ vs 10^9^ were 0.034, 0.001, and 0.002, respectively. (**B**) Approximately 5 × 10^7^, 10^8^, 5 × 10^8^, and 10^9^ Δ*phnCDE* bacteria were plated on TGPn plates. Agar plugs were collected at time 0 and after 96 h of incubation at 37°C. Bacteria were eluted from the plugs, diluted in saline, and plated on L-agar. The number of bacteria on each plate was estimated from the CFU counts in each plug, scaled to the total plate area. Three plugs were collected per plate at each time point. Each data point represents the mean ± standard deviation of the total bacterial count per plate. Welch *t*-test analyses showed statistically significant differences (*P* < 0.05) for each bacterial concentration between 0 and 96 h, except for 10^8^ Δ*phnCDE*, which gave a *P*-value of 0.054.

To assess the frequency of *phn^+^* revertants, approximately 5 × 10^7^, 10^8^, 5 × 10^8^, and 10^9^ MG1655 (*phn^−^*) cells were plated on TGPn medium and incubated for 4 days at 37°C. Revertant frequencies ranged from 8.6 × 10^−7^ to 1.1 × 10^−5^, representing a 12.8-fold variation depending on the initial inoculum size, with lower numbers of plated cells yielding higher apparent mutant frequencies (Fig. 1A). This discrepancy can be explained by the growth of wild-type cells on TGPn plates fueled by trace Pi contamination in AmPn, which inflated the final bacterial counts.

To test this possibility, we examined whether Δ*phnCDE* bacteria could grow on TGPn plates. A total of 5 × 10^7^, 10^8^, 5 × 10^8^, and 10^9^ Δ*phnCDE* cells were plated on TGPn medium. Agar plugs were collected at time 0 and after 96 h of incubation. The bacteria were eluted from the plugs, serially diluted, and plated on L-agar. As shown in Fig. 1B, the number of bacterial cells increased in all plates after 96 h. Overall, the lower the initial bacterial load, the greater the fold increase in cell numbers (Table 1). While bacteria seeded at a density of approximately 4 × 10^7^ cells grew for about six generations (a 63-fold increase), those plated at an initial concentration of ∼4 × 10^9^ cells replicated only for 2.1 generations (a fourfold increase in population size). This was expected, since the carrying capacity of the plates was identical and only the initial inoculum size varied. These results suggest that the frequency of *phn^+^* revertants was artificially inflated by bacterial growth on the selective plates due to residual phosphate contaminants.

**TABLE 1:** Growth of Δ*phnCDE* on TGPn plates

Bacteria (0 h)	Bacteria (96 h)	Fold increase^*a*^	Generations^*b*^
4.13 × 10^7^	2.61 × 10^9^	63	6.0
8.87 × 10^7^	2.49 × 10^9^	28	4.8
3.65 × 10^8^	1.57 × 10^10^	43	5.4
3.64 × 10^9^	1.53 × 10^10^	4	2.1

^*a*^ $Fold\;increase=\frac{{bac}_{96}}{{bac}_{0}}$.

^*b*^ $No.\;of\;generations=\frac{log({bac}_{96})-log({bac}_{0})}{log(2)}$.

**Fig 2:**
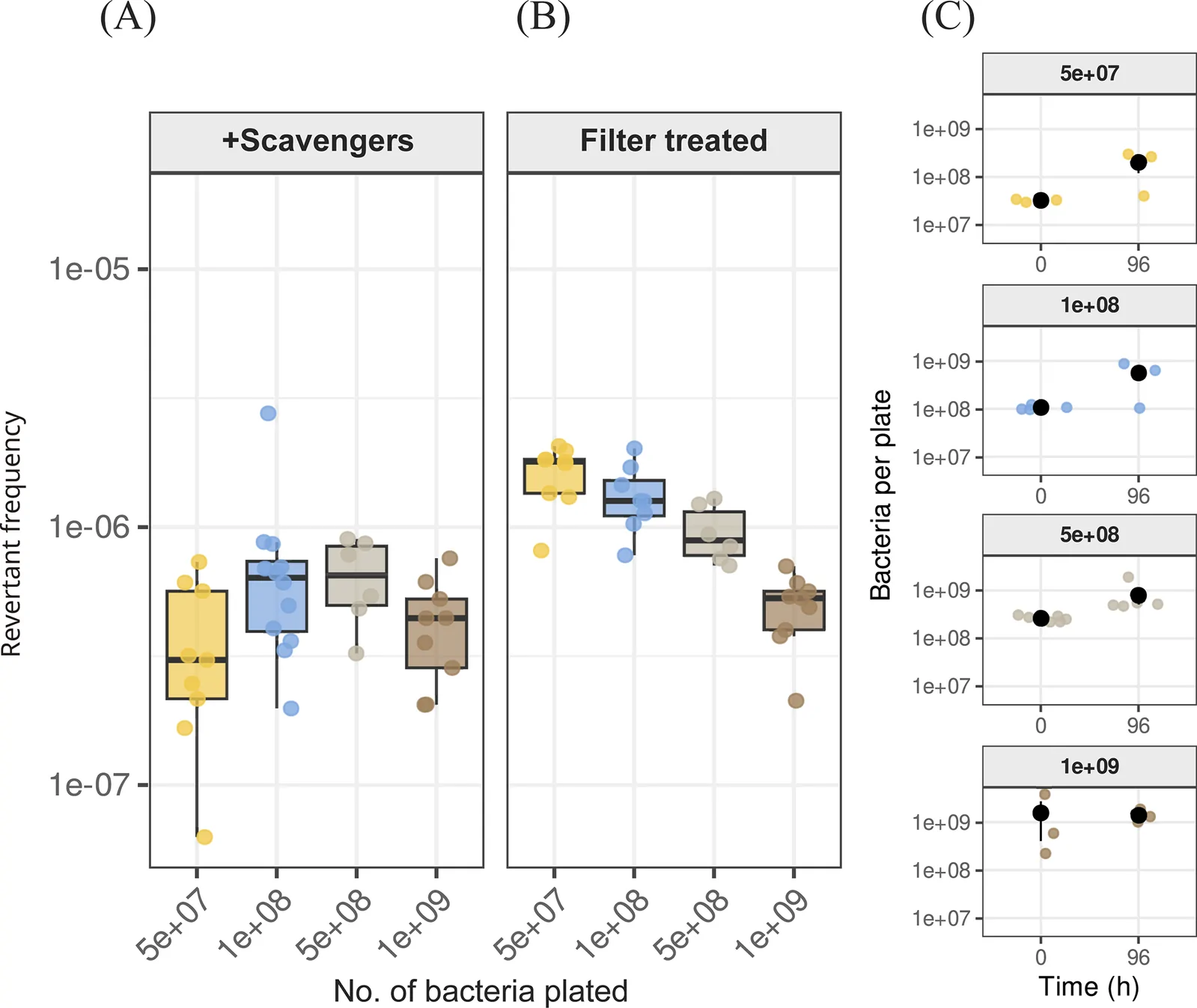
Frequency of *phn^+^* revertants in MG1655 cultures containing 5 × 10^7^, 10^8^, 5 × 10^8^, or 10^9^ cells: (**A**) plated in the presence of 10^9^ Δ*phnCDE* scavenger cells (ANOVA revealed no statistically significant differences among the groups); (**B**) plated on TGPn medium previously treated with membrane filters containing Δ*phnCDE* cells to remove phosphate contaminants. Statistically significant differences (ANOVA followed by Tukey’s *post hoc* test) were observed for the following comparisons: 5 × 10^7^ vs 5 × 10^8^ (*P* = 0.02), 5 × 10^7^ vs 10^9^ (*P* < 0.001), 10^8^ vs 10^9^ (*P* < 0.001), and 5 × 10^8^ vs 10^9^ (*P* = 0.043); (**C**) growth of Δ*phnCDE* cells on TGPn plates under the same conditions. Welch *t*-tests revealed no significant differences between 0 h and 96 h in all pairwise comparisons. All plates were incubated for 96 h at 37°C. Bacteria processing and counting and revertant frequencies were determined as described in the legend to Fig. 1.

A common strategy to minimize the effect of contaminants on selective plates for non-lethal mutational systems, such as the selection of *lac* mutants, is to co-plate the target culture with scavenger bacteria (e.g., Δ*lacZ* cells for *lac* mutant selection [14]). These scavenger cells eliminate residual nutrient sources that could otherwise support unwanted growth on the selective medium. Following this approach, we plated varying concentrations of MG1655 cells in the presence of 10^9^ Δ*phnCDE* cells on TGPn plates. As shown in Fig. 2, the frequency of *phn^+^* revertants remained relatively constant, ranging from 3.6 × 10^−7^ to 7.5 × 10^−7^ (mean = 5.46 × 10^−7^), regardless of the initial bacterial density. These values are comparable to the lowest RF observed in the absence of scavenger cells (8.6 × 10^−7^). It seems, thus, that the plating of scavenger cells eliminated the problem of wild-type bacteria growth on the selective plates. However, there is still the possibility that high bacterial density might be inhibiting the emergence of *phn^+^*, as shown for other mutational systems (11, 12), and that the real RF is being underestimated.

To overcome the issue of *phn^−^* bacteria growth on the selective plates with Pi contaminants and, on the other hand, to prevent excessive cell density, we tested an alternative strategy: 10^9^ Δ*phnCDE* cells were applied onto membrane filters placed on top of TGPn plates and incubated for 48 h to consume the remaining Pi contaminants. It is worth noting that all added medium components, including glucose, two aminoethylphosphonic acids, and other salts, are present in excess in the selective plates. Following incubation, the filters were removed, and the plates were then seeded with MG1655 cells to assess the frequency of *phn^+^* revertants. The frequency of revertants now ranged from 4.9 × 10^−7^ to 1.6 × 10^−6^ and showed a negative correlation with the concentration of plated bacteria, although the magnitude of the effect of cell density was considerably smaller than that observed in cells plated on non-treated TGPn plates. The RF in plates containing 5 × 10^7^ bacteria was 3.3-fold higher than that observed in plates seeded with 10^9^ bacteria, whereas in the non-treated set, this difference reached 12.8-fold (Fig. 1A). The reduced RF observed at high plating densities may reflect density-dependent inhibition. Alternatively, the higher RF observed on low-density plates may simply reflect the growth of wild-type cells on the selective medium.

To verify whether treatment with scavenger cells on membrane filters effectively removed Pi contaminants, we attempted to measure the Pi concentration in plates treated with membrane filters, as described in section Microdetermination of Pi. Quantitative assessment proved unfeasible because Pi was retained by the agar itself (Fig. S1A). Nevertheless, qualitative differences were evident: the untreated medium displayed a bluish coloration characteristic of Pi, whereas the solid medium treated with the scavenger filter remained uncolored, indicating that the Pi concentration was below the detection limit (Fig. S1B). Furthermore, when approximately 250 Δ*phnCDE* bacteria were spread onto the treated plates and incubated for 24 h, no colonies were observed. In contrast, the untreated plates yielded readily visible colonies (Fig. S1C). These results indicate that Pi was largely, if not entirely, removed by the scavenger filters. However, the definitive test of the scavenger-filter treatment was to determine whether it effectively prevented bacterial growth on plates previously subjected to Pi removal. For this purpose, increasing numbers of Δ*phnCDE* cells were seeded onto treated plates and incubated at 37°C. Agar plugs were collected at 0 and 96 h, the bacteria were eluted, diluted, and plated on L-agar for CFU counting. Figure 2C shows that Δ*phnCDE* density after 96 h increased 6, 5, and 3-fold in plates initially containing 5 × 10^7^, 10^8^, and 5 × 10^8^ cells, respectively, but did not increase in plates seeded with 10^9^ bacteria. These results indicate that treatment of TGPn plates with membrane filters containing Δ*phnCDE* cells effectively removed most, but not all, Pi contaminants (compare the fold increase in growth with those observed in non-treated plates in Table 1).

By accounting for the fold increase in bacterial abundance observed for Δ*phnCDE* cells (i.e., the number of times the population increased over 96 h on residual Pi), it is in principle possible to correct the nominal inoculum to estimate the actual number of MG1655 cells present on the selective plates. For example, plates nominally seeded with 5 × 10^7^ MG1655 cells (first row of Table 2) supported growth to approximately 4.1 × 10^7^ × 6 = 2.46 × 10^8^ cells.

**TABLE 2:** Frequency of revertants in selective media treated with scavenger filters

Plated bacteria	Frequency^*a*^	Fold increase^*b*^	Corrected frequency^*c*^
4.1 × 10^7^	1.64 × 10^−6^	6.21	2.64 × 10^−7^
1.3 × 10^8^	1.33 × 10^−6^	5.26	2.54 × 10^−7^
4.1 × 10^8^	9.61 × 10^−7^	3.01	3.19 × 10^−7^
1.3 × 10^9^	4.92 × 10^−7^	0.90	5.44 × 10^−7^
Mean	1.11 × 10^−6^	–^*d*^	3.45 × 10^−7^

^*a*^ Frequency = RF.

^*b*^ Fold increase represents the increase in Δ*phnCDE* population on TGPn filter-treated plates as shown in Fig. 2C.

^*c*^ Corrected frequency was calculated by multiplying the number of plated bacteria by the respective fold increase and using this product as *N* in $freq=\frac{mutants}{N}$.

^*d*^ –, not applicable.

Consequently, the actual RF should be sixfold lower, that is, 2.64 × 10^−7^. The average corrected value of the *phn^−^* RF was 3.45 × 10^−7^ (Table 2), close to that obtained with the method in which scavenger Δ*phnCDE* cells were plated together with MG1655 (5.46 × 10^−7^).

In summary, the *phn^+^* reversion frequency depends on the number of bacteria plated on the selective medium, which is influenced by the presence of Pi contaminants in the phosphonate source. This issue can be mitigated either by directly plating an excess of Δ*phnCDE* bacteria to scavenge the excess Pi or by applying Δ*phnCDE* cells on a filter membrane prior to plating MG1655 (*phn^−^* cells carrying the 8 bp insertion in *phnE*). However, the latter approach does not completely prevent MG1655 growth on the selective plates. A correction can therefore be applied by accounting for the fold increase in bacterial numbers on the selective medium.

The mean reversion frequencies obtained with these methods were 1.11 × 10^−6^ (plates pre-treated with scavenger filters), 5.46 × 10^−7^ (plates directly seeded with scavengers), and 3.45 × 10^−7^ (after correction applied to pre-treated plates). Applying the growth correction to the RF in untreated plates (shown in Fig. 1) yielded a mean reversion frequency of 1.16 × 10^−7^.

Once most of the Pi was removed from the selective plates, the negative correlation between RF and cell density became less pronounced (compare Fig. 1A and 2B). Moreover, the corrected frequency values (Table 2) closely matched those obtained from plates seeded with 10^9^ scavenger cells. Altogether, these results suggest that cell density did not inhibit the frequency of *phn^+^* revertants.

### Relative fitness of the *phn^−^* mutant

To determine whether the *phn^+^* allele confers a selective advantage over its *phn^−^* counterpart, strains MG1655 (*phnE*::8-bp octamer) Δ*lacZ* and MG1655 *phn^+^* were competed in a Pi-limited chemostat. Equal inocula of each strain were introduced into a culture flask containing TGP minimal medium with 50 µM KH_2_PO_4_ (the limiting nutrient) and maintained for 48 h under steady-state conditions. Samples withdrawn during the experiment were plated on L-agar supplemented with X-gal to distinguish the competitors. The selection coefficient of strain MG1655 *phn^+^* was −0.0165 ± 0.009 (mean ± SE of six independent chemostat competitions), indicating a fairly small advantage for the *phn^−^* mutant over the *phn^+^* strain under these conditions. To illustrate the competition dynamics, Table S1 shows the proportion of *phn^+^* bacteria at each time point. To verify that the *phn^−^* allele in MG1655 Δ*lacZ* had not reverted to *phn^+^*, several white (*lacZ^−^*) and blue (*phn^+^*) colonies were tested for growth on TGPn plates. Unlike the blue colonies, all white (*phn^−^*) colonies failed to show consistent growth, confirming that no revertants were present in the chemostat. Instead, a few colonies that correspond to spontaneous *phn^+^* revertants that emerged from the bacteria streaked on the plate (the plate was not treated with Δ*phnCDE* filters) could be spotted (Fig. S2).

### Distribution of the *phn^−^* allele across *E. coli* strains

To find out the prevalence of the 8 bp octamer in *phnE* across *E. coli* strains, we searched 4,501 complete genomes in GenBank for *phnE* mutants. We employed two complementary strategies to ensure that all genomes carrying the insertion were identified while avoiding duplicate entries: (i) searching for *phoE* pseudogenes in the .gff annotation files, and (ii) searching for the 8 bp octamer within the *phoE* gene sequences in the .fna files. Nine hundred and fourteen genomes were classified as *phn^−^*. Among these, 904 carried the characteristic 8 bp insertion in *phnE*, while 10 harbored other nonsense mutations. Of the *phn^−^* genomes, 264 corresponded to laboratory strains, most of them derived from the original K-12 lineage; 450 were clinical isolates; and 200 were environmental isolates (veterinary strains were grouped with environmental isolates, whereas human-derived strains were classified as clinical isolates).

The list of strains is provided in Table S2. After excluding the 264 laboratory strains—most of which derive from a single propagated K-12 ancestor—the frequency of *phn^−^ E. coli* lineages reaches 15.3% in this data set. This unexpectedly high prevalence suggests that the 8-bp insertion in *phnE* may confer a selective advantage to *E. coli* in certain environments, rather than representing a neutral or deleterious mutation.

## DISCUSSION

Non-lethal mutagenicity systems, such as *phn^−^* reversion, *lac^c^* selection on P-gal (14), *lacZ* reversions (15), and many others, share a common issue: non-mutant growth on selective plates driven by media impurities, which inadvertently increases the number of wild-type bacterial generations even after exposure to the selection agent. In the *lacZ* and *lacI* systems, this problem was addressed by plating scavenger bacteria that are unable to grow in the presence of the selection agent (14, 17). However, introducing additional cells can interfere with the emergence of true mutants (11, 12), thereby masking the actual mutant frequency.

One way to circumvent this issue is to use high-purity reagents, such as agarose instead of agar (14). However, this approach substantially increases experimental costs, given that agarose is considerably more expensive, and it may not be feasible for other mutational systems in which comparably pure reagents are unavailable. Another possibility is to seed plates with scavenger cells placed on membrane filters that are removed after pre-incubation at the appropriate temperature. The advantage of this approach is that scavenger bacteria do not remain on the plate, where they could otherwise inhibit the growth of genuine mutants. Our results suggest that this method was only partially effective, as the Δ*phnCDE* strain was still able to grow for a few generations on the treated plates. However, statistical analyses showed that, unlike the untreated plates (Fig. 1B), no statistically significant differences in growth between 0 h and 96 h were detected at any initial bacterial concentration when filtered-treated plates were used (Fig. 2C). This suggests that growth on the treated plates was not as straightforward as it might seem and raises two possibilities: the filter membranes may have been more effective than anticipated, or the experiment may not have had enough statistical power to detect subtle differences in growth yield. It should be noted, however, that the residual Pi levels were very low (Fig. S1).

These observations indicate that the scavenger-filter treatment was highly effective at removing contaminating phosphate. Nonetheless, trace amounts of Pi remained sufficient to support limited growth of the Δ*phnCDE* mutant for a few generations on the treated plates.

Even the highest RF we observed, 1.64 × 10^−6^ (obtained by plating 4.1 × 10^7^ bacteria on treated Phn plates), was still an order of magnitude lower than the value reported by Guillemet and Moreau (10), and more than four orders of magnitude lower than those observed by Iqbal et al. (9). Guillemet and Moreau (10) reported a *phn^+^* revertant frequency of 2 × 10^−5^ in strain ENZ535 (MG1655), although no methodological details were provided on how this estimate was obtained. In contrast, Iqbal et al. (9) reported substantially higher frequencies, claiming that *phn^+^* revertants arose at rates exceeding 10^−2^ in the K-12 strains DH5-α and MC4100. In these tests, stationary-phase bacteria were washed and incubated for 10 days at 37°C on plates containing methyl phosphonate as the P source. The authors did not mention using scavenger cells or report the density of bacteria plated. However, obtaining a reversion frequency of >10^−2^ would require plating no more than a few hundred cells. Under such conditions, wild-type bacteria would likely have grown on the selective plate by exploiting trace amounts of Pi, artificially inflating the apparent RF. The particular care taken in the current study to eliminate Pi traces from the selective plates likely resulted in a more accurate RF estimate for *phn^+^* than previously reported. The main limitation of this study, however, was the lack of experimental evidence regarding the molecular mechanism underlying *phn^−^* reversion.

Gene loss in natural isolates is not restricted to *phnCDE*. For example, *E. coli* natural isolates have been reported to carry *rpoS* knockout mutations or mutations that strongly reduce *rpoS* expression or function, albeit at a lower frequency of approximately 1.8% (18). Similarly, in *Salmonella* Typhimurium, mutants deficient in the glycerol-3-phosphate antiporter *glpT* have been shown to be positively selected in nature, clinical, and laboratory settings (19). Also in *Salmonella*, 84 genes associated with virulence, motility, and chemotaxis, biofilm formation, and antibiotic resistance were identified whose inactivation appears to be positively selected in natural populations (20). Interestingly, neither *phnE* nor any other gene in the *phn* operon is among the 84 genes reported to undergo frequent inactivation in this bacterium.

The fitness of the *phn^+^* strain under Pi-limiting conditions was not higher than that of the *phn^−^* strain, suggesting that even under conditions expected to favor an intact *phnCDE* operon (the PhnCDE transport system may take up Pi in addition to Pn [6]), no selective advantage of this allele was detected. This may help explain the relatively high proportion of *phn^−^* bacteria observed in natural environments in the *in silico* analyses. The finding is consistent with the idea that the PhnCDE system does not confer a meaningful fitness advantage in environments that are not rich in phosphonates. Under these conditions, mutation of *phnE* may represent a case of antagonistic pleiotropy, whereby loss of function is selectively favored despite the gene’s potential benefit in more restricted ecological niches.

## MATERIALS AND METHODS

### Bacterial strains and media

MG1655 is the prototype *E. coli* K-12 strain. MG1655 derivatives used in this study were strain MC01 (MG1655 Δ*lacZ*), RS02 (MG1655 Δ*phnCDE*::Km^R^ [6]), and RS03 (MG1655 *phn^+^* [6]). Lysogeny Broth (LB) is as described (21). T-salts (11 mM glucose, 0.12 M Tris-HCl, 80 mM NaCl, 20 mM KCl, 20 mM NH_4_Cl, 0.98 mM MgCl₂·6H₂O, 2.46 mM Na_2_SO_4_, 2 mM CaCl_2_, 2 μM FeCl_3_, 2 μM ZnCl_2_, pH 7.5)-based media TGP and TGPn contain 1 mM KH_2_PO_4_ or 1 mM 2-aminoethylphosphonic acid, respectively.

### Selection of *phn^+^* revertants

To select *phn^+^* revertants, *E. coli* K-12 MG1655 cells were grown overnight in TGP minimal medium at 37°C with shaking. The cultures were washed with saline, and dilutions containing the desired number of cells were plated on TGPn minimal medium, which uses 2-aminoethylphosphonic acid as the sole phosphorus source. In parallel, aliquots were diluted and plated on L-agar to determine the exact number of viable cells applied to each plate. After 96 h of incubation at 37°C, plates were examined, and the mutant frequency was calculated by dividing the number of revertant colonies by the total number of plated bacteria (estimated from the LB counts).

Trace phosphate was removed using phosphate-scavenging bacteria in two ways. (i) Selective medium plates were pre-seeded with 10^9^ cells of strain RS02 (MG1655 Δ*phnCDE*::Km^R^) and incubated for 2 h before plating MG1655. (ii) Alternatively, sterile cellulose-ester membranes (90 mm diameter, 0.45 µm pores) were placed on the selective plates, and 10^9^ RS02 cells were spread on top. The plates were incubated for 48 h at 37°C, after which the membranes were removed and MG1655 cultures were plated onto the treated medium.

### Microdetermination of Pi

The amount of contaminating Pi in the working solutions used to prepare the culture medium was measured using the method described by (22). The following solutions were prepared in advance: 167 mM sulfuric acid, 2.5% ammonium molybdate, and 10% ascorbic acid. The working reagent was freshly prepared at the time of the assay by mixing one volume of 167 mM sulfuric acid, one volume of 2.5% ammonium molybdate, one volume of 10% ascorbic acid, and two volumes of deionized water. The sample to be tested was incubated with an equal volume of the working reagent at 37°C for 1 h. Absorbance at 820 nm was measured using a spectrophotometer. The contaminating Pi concentration was calculated using the OD${}_{820}$ value of the sample and the calibration curve equation obtained from standards containing 2.5, 5, 10, 20, 40, and 80 µM KH_2_PO_4_.

### Chemostat competition

Strains MG1655 *phn^+^* and MG1655 *phn^−^* Δ*lacZ* were competed under phosphate-limiting conditions. Pre-cultures were prepared by inoculating single colonies of each strain into 6 mL of TGP defined medium supplemented with 1 mM KH_2_PO_4_, followed by overnight incubation at 37°C with orbital shaking at 180 rpm. Culture turbidity was measured at OD_600_, and equal cell numbers of each strain were mixed at a 1:1 ratio. The mixed culture was used to inoculate a chemostat containing TGP medium with limiting Pi (0.05 mM KH_2_PO_4_) to a final concentration of approximately 8 × 10^7^ bacteria/mL. Cultures were grown at 37°C under steady-state conditions with a dilution rate of 0.5 h^−1^ for 48 h. Time zero was defined as 3 h after inoculation. At this time point, a 3 mL sample was collected, serially diluted in 0.9% NaCl, and plated on L-agar supplemented with X-gal and IPTG to allow discrimination between strains. Additional samples were collected at 24 h and 48 h and processed identically. Relative fitness was quantified by calculating the selection coefficient. For each time point (0, 24, and 48 h), the ratio of competing strains was determined, and the natural logarithm of this ratio, ln(*phn*^+^/*phn*^−^), was plotted as a function of time. The slope of the resulting linear regression corresponds to the selection coefficient (23). The competitions were conducted in six independent experiments.

To verify that the MG1655 *phn^−^* Δ*lacZ* strain did not revert to *phn^+^* via deletion of the 8 bp insertion in *phnE* during the competition, several white (*lacZ*-negative) colonies recovered from the 48 h plates were isolated and streaked onto TGPn plates. Figure S2 shows representative plates of bacteria subjected to chemostat competition and subsequently streaked on TGPn. All colonies that were originally *phn^+^* retained the ability to grow on phosphonate, whereas none of the originally *phn^−^* colonies acquired growth on phosphonate during the competition.

### Bioinformatics

The genome files (.fna and .gff) of 4,501 complete *E. coli* genomes were downloaded from ftp.ncbi.nlm.nih.gov/genomes/refseq/bacteria/. To identify genomes carrying null (nonsense or frameshift) mutations in *phnE*, we used two complementary approaches: (i) screening the .gff files for “pseudogene” annotations in the *phnE* CDS using an *awk* script, and (ii) scanning the .fna files for the 8 bp ABB octameric repeat using the fuzznuc (EMBOSS suite). To classify each strain as laboratory, clinical, or environmental, the GenBank file of each of the 914 genomes was manually inspected.

### Statistical analysis

ANOVA was followed by either Tukey’s or Games-Howell *post hoc* tests, depending on whether the data passed Levene’s test for equality of variances. Welch’s *t*-tests were used for pairwise comparisons of bacterial growth in agar plugs (0 h vs 96 h). All statistical analyses were performed using JASP (24).

## SUPPLEMENTAL MATERIAL

Supplemental figures and tables jb.00073-26-s0001.pdf Figures S1 and S2, and Tables S1 and S2.

ASM does not own the copyrights to Supplemental Material that may be linked to, or accessed through, an article. The authors have granted ASM a non-exclusive, world-wide license to publish the Supplemental Material files. Please contact the corresponding author directly for reuse.
