# Supplementary material for: phnE frameshift reversion frequency: beyond density effects to genomic prevalence
Source: J Bacteriol. 2026 Jul 14;208(8):e00073-26. doi: 10.1128/jb.00073-26 (PMC13492104; doi:10.1128/jb.00073-26)
Supplement: Supplementary file 1 [file jb.00073-26-s0001.pdf]

SUPPLEMENT

phnE Frameshift Reversion Frequency: Beyond Density Effects to Genomic Prevalence

Luísa Andrea Villanueva da Fonseca[1], Marina Caldas Leite[1], and Beny Spira[1]*

[1]Departamento de Microbiologia, Instituto de Ciências Biomédicas Universidade de São Paulo, São Paulo-SP, Brazil

*Corresponding author, email: benys@usp.br

Supplement

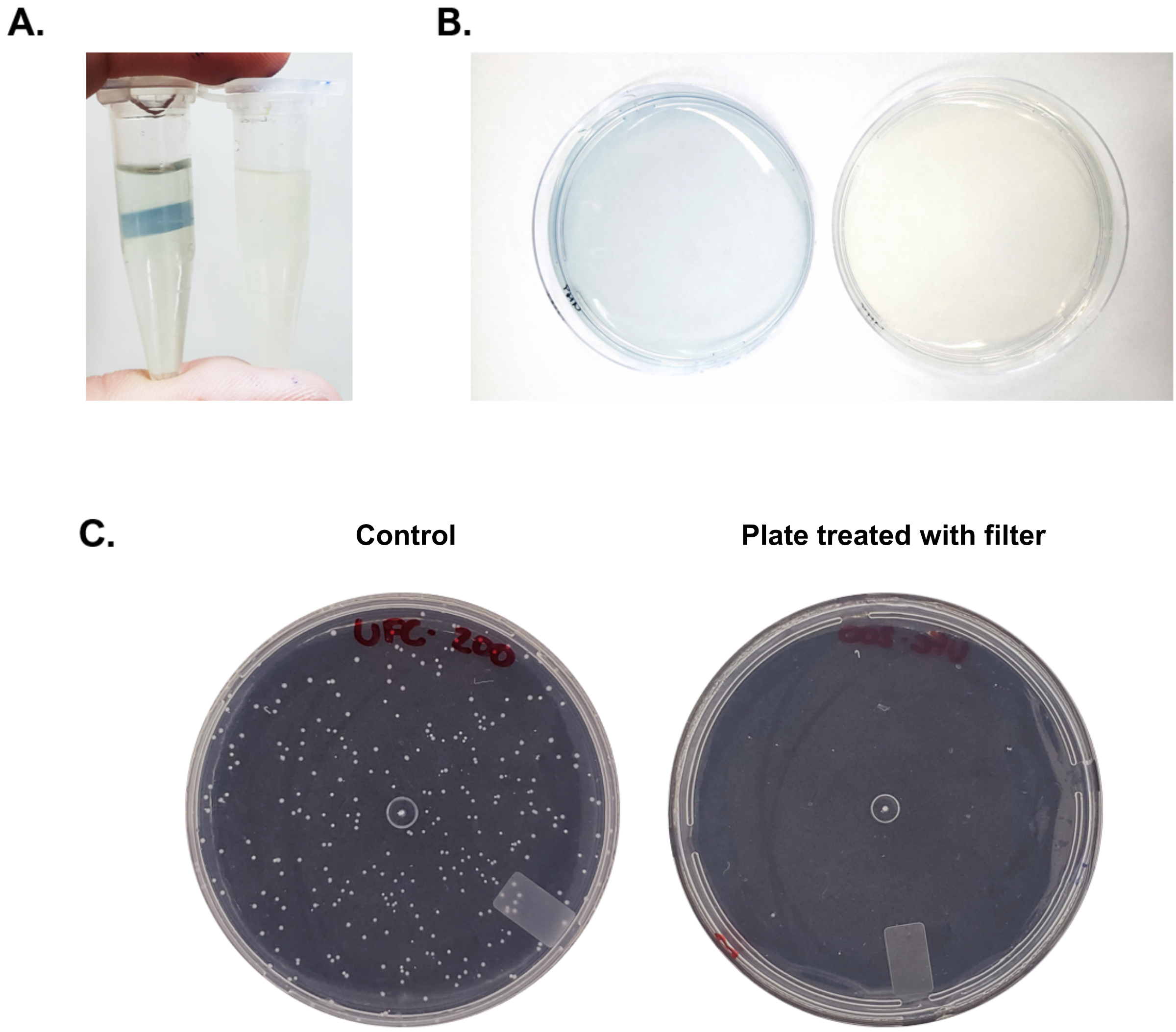

Fig S1. Assessment of the presence of Pi in TGPn plates treated with filter membranes. **A.** Microdetermination of Pi by the molybdate method in plugs removed from the culture medium. Left tube, TGPn agar from a non-treated plate; right tube, TGPn agar from a filter-treated plate, Pi is retained in the agar. **B.** The reagent used for determining Pi in the medium was poured onto TGPn plates treated with membrane (no pigmentation) and untreated plates (bluish colour), again showing that TGPn plate treated with the filter membranes removes most Pi from the medium. **C.** Approximately 250 Δ*phnCDE* cells were spread onto an untreated TGPn plate (Control) and onto a TGPn plate previously treated with membrane-based scavengers (Plate treated with filter), no colony formation is observed on the treated TGPn plate, indicating that the treated medium carries considerably less Pi than the untreared medium.

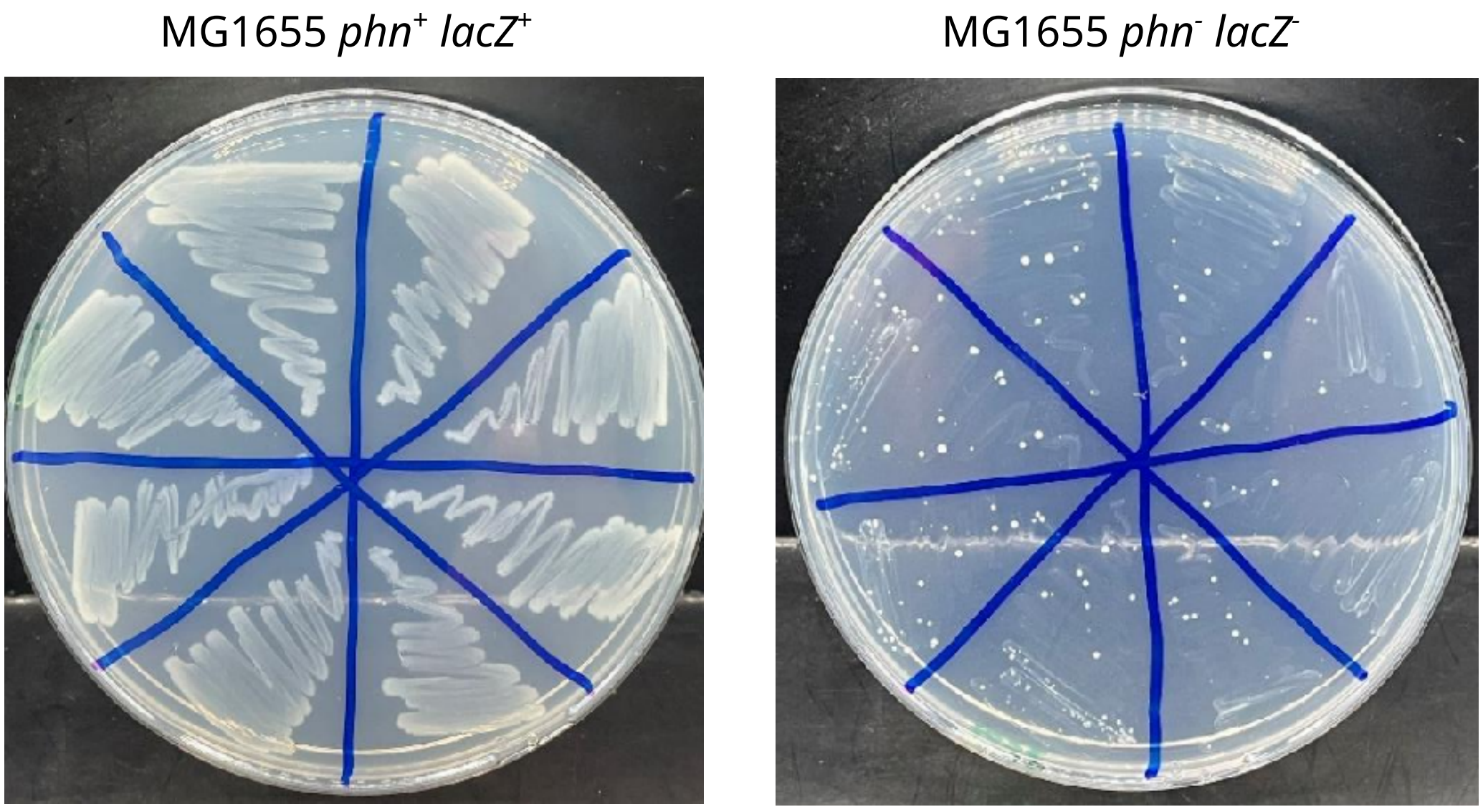

Fig S2. Eight white colonies (MG1655 *phn*$^-$ *lacZ*) and 8 blue colonies (MG1655 *phn*$^+$ *lacZ*$^+$), isolated after 48 h of competition in a chemostat, were streaked onto TGPn plates and incubated for 72 h. All *lacZ*$^+$ bacteria retained the ability to grow on phosphonate, indicating that the *phn*$^+$ gene was maintained. In contrast, *lacZ* cells were unable to grow on TGPn plates, except for a small number of colonies that grew on the plate (using Pi traces) and reverted the *phn*$^-$ phenotype.

Table S1. Competition dynamics: proportion of *phn*$^+$ cells in the chemostat.

Time (h)	Mean % of *phn*$^+$ ±SE
3	40.72% ± 2.60%
24	31.24% ± 5.50%
48	28.87% ± 7.28%

Table S2. Classification of the genomes carrying a *phnE* mutation according to the strain name and source (clinical, environmental or laboratory).

**Genome**	**Strain name**	**Source**	**Genome**	**Strain name**	**Source**
NC_017633.1	ETEC	Clinical isolate	NZ_AKBV01001.1	K-12 sub	Lab strain
NZ_CP051001.1	98-3133	Clinical isolate	NC_017638.1	DH1	Lab strain
NZ_CP016182.2	EC590	Clinical isolate	NZ_AKVX01001.1	K-12 sub	Lab strain
NZ_CP018103.1	MRSN352231	Clinical isolate	NC_022364.1	LY180	Lab strain
NZ_CP018115.1	MRSN346638	Clinical isolate	NZ_HG738867.1	K-12 sub	Lab strain
NZ_CP018121.1	MRSN346355	Clinical isolate	NZ_CP009273.1	K-12 sub	Lab strain
NZ_CP018109.1	MRSN346595	Clinical isolate	NZ_CP009644.1	K-12	Lab strain
NZ_CP019213.3	WCHEC050613	Clinical isolate	NZ_CP009789.1	K-12	Lab strain

Genome	Strain name	Source	Genome	Strain name	Source
NZ_CP021175.1	5CRE51	Clinical isolate	NZ_CP009685.1	K-12 sub	Lab strain
NZ_CP021532.1	AR_0149	Clinical isolate	NZ_LM995446.1	K-12	Lab strain
NZ_CP021683.1	AR_0162	Clinical isolate	NZ_LM993812.1	K-12	Lab strain
NZ_CP021691.1	AR_0151	Clinical isolate	NZ_CP010438.1	K-12	Lab strain
NZ_CP021732.1	AR_0114	Clinical isolate	NZ_CP010439.1	K-12	Lab strain
NZ_CP021736.1	AR_0150	Clinical isolate	NZ_CP010440.1	K-12	Lab strain
NZ_CP021879.1	AR_0137	Clinical isolate	NZ_CP010441.1	K-12	Lab strain
NZ_CP023201.1	360/16	Clinical isolate	NZ_CP010442.1	K-12	Lab strain
NZ_CP023870.1	FDAARGOS_434	Clinical isolate	NZ_CP010443.1	K-12	Lab strain
NZ_CP024830.1	CREC-532	Clinical isolate	NZ_CP010444.1	K-12	Lab strain
NZ_CP024859.1	AR_0014	Clinical isolate	NZ_CP010445.1	K-12	Lab strain
NZ_CP024815.1	CREC-629	Clinical isolate	NZ_LN832404.1	K-12 sub	Lab strain
NZ_CP024855.1	AR_0011	Clinical isolate	NZ_CP011342.2	K-12 sub	Lab strain
NZ_CP025950.3	SCEC020023	Clinical isolate	NZ_CP011343.2	K-12 sub	Lab strain
NZ_CP024134.1	14EC017	Clinical isolate	NZ_CP011495.1	NCM3722	Lab strain
NZ_CP019051.1	CRE1540	Clinical isolate	NZ_CP012125.1	DH1Ec095	Lab strain
NZ_CP019071.1	CRE1493	Clinical isolate	NZ_CP012126.1	DH1Ec104	Lab strain
NZ_CP025627.1	SCEC020007	Clinical isolate	NZ_CP012127.1	DH1Ec169	Lab strain
NZ_CP026199.1	ECONIH6	Clinical isolate	NZ_CP011113.2	RR1	Lab strain
NZ_CP026580.3	WCHEC005237	Clinical isolate	NZ_CP012868.1	K-12 sub	Lab strain
NZ_CP027355.1	2013C-4991	Clinical isolate	NZ_CP012869.1	K-12 sub	Lab strain
NZ_CP028310.1	APEC	Clinical isolate	NZ_CP012870.1	K-12 sub	Lab strain
NZ_CP028578.2	WCHEC005784	Clinical isolate	NZ_CP014225.1	K-12 sub	Lab strain
NZ_CP029115.1	AR435	Clinical isolate	NZ_CP014270.1	K-12	Lab strain
NZ_CP034595.1	WCHEC035053S1G0	Clinical isolate	NZ_CP014272.1	K-12	Lab strain
NZ_CP022664.1	FORC	Clinical isolate	NZ_CP014348.1	JW5437-1 sub	Lab strain
NZ_CP042893.1	CFSAN061772	Clinical isolate	NZ_CP016018.1	ER1821R	Lab strain
NZ_CP036177.1	WCHEC025970	Clinical isolate	NZ_CP017100.1	K-12	Lab strain
NZ_CP029057.1	FORC_081	Clinical isolate	NZ_CP017979.1	K-12 sub	Lab strain
NZ_CP042336.1	GZ04-0086	Clinical isolate	NZ_CP018801.1	tolC-	Lab strain
NZ_CP038453.1	EC-129	Clinical isolate	NZ_CP025268.1	K-12 sub	Lab strain
NZ_CP041392.1	ECOL-18-VL-LA-PA-Ryan-0026	Clinical isolate	NZ_CP025520.1	DH5alpha	Lab strain
NZ_CP043217.1	EC-107	Clinical isolate	NZ_CP027060.1	K-12 sub	Lab strain
NZ_CP043185.1	PG20180171	Clinical isolate	NZ_CP028702.1	J53	Lab strain
NZ_CP043183.1	PG20180172	Clinical isolate	NZ_CP031214.1	C600	Lab strain
NZ_CP043187.1	PG20180170	Clinical isolate	NZ_CP032667.1	K-12 sub	Lab strain
NZ_CP043189.1	PG20180175	Clinical isolate	NZ_CP030240.1	ER1709	Lab strain
NZ_CP043191.1	PG20180173	Clinical isolate	NZ_CP040667.1	S17-1	Lab strain
NZ_CP043193.1	PG20180063	Clinical isolate	NZ_CP042867.1	ATCC	Lab strain
NZ_CP043195.1	PG20180062	Clinical isolate	NZ_CP042865.1	ATCC	Lab strain
NZ_CP043197.1	PG20180061	Clinical isolate	NZ_CP045457.1	ET12567	Lab strain
NZ_CP043201.1	PG20180059	Clinical isolate	NZ_CP045741.1	DH5alpha	Lab strain
NZ_CP043199.1	PG20180060	Clinical isolate	NZ_CP047074.1	lacZ_1	Lab strain
NZ_CP043205.1	PG20180054	Clinical isolate	NZ_CP047075.1	lacZ_2	Lab strain
NZ_CP043203.1	PG20180058	Clinical isolate	NZ_CP047076.1	lacZ_3	Lab strain
NZ_CP043207.1	PG20180053	Clinical isolate	NZ_CP047077.1	pdeL_1	Lab strain
NZ_CP043209.1	PG20180052	Clinical isolate	NZ_CP047078.1	pdeL_2_1	Lab strain
NZ_CP043211.1	PG20180050	Clinical isolate	NZ_CP047079.1	pdeL_2_2	Lab strain
NZ_CP043213.1	CV601gfp	Clinical isolate	NZ_CP047127.1	K-12	Lab strain
NZ_CP043227.1	Ec-050	Clinical isolate	NZ_CP053603.1	Dam/Dcm	Lab strain
NZ_CP041627.1	ETEC6	Clinical isolate	NZ_CP053604.1	NEB10-beta	Lab strain
NZ_CP043946.1	AR202.2	Clinical isolate	NZ_CP053605.1	NEB_Turbo	Lab strain
NZ_CP043942.1	AR216.2b	Clinical isolate	NZ_CP053607.1	NEB5-alpha_F'Iq	Lab strain
NZ_CP045277.1	LAU-OXA	Clinical isolate	NZ_CP060708.1	MFDpir	Lab strain

Genome	Strain name	Source	Genome	Strain name	Source
NZ_CP043486.1	SL112	Clinical isolate	NZ_CP064677.1	BW25113	Lab strain
NZ_CP041955.1	EC2	Clinical isolate	NZ_CP068706.1	J53-p1-pV-hybrid-1	Lab strain
NZ_CP026929.2	CFS3246	Clinical isolate	NZ_CP069132.1	BW25113 sub	Lab strain
NZ_CP026932.2	CFS3273	Clinical isolate	NZ_CP069133.1	BW25113 sub	Lab strain
NZ_CP026935.2	CFS3292	Clinical isolate	NZ_CP069134.1	BW25113 sub	Lab strain
NZ_AP021893.1	2018-06-4CC	Clinical isolate	NZ_CP072661.1	BW25113	Lab strain
NZ_AP021894.1	2018-10-1CC	Clinical isolate	NZ_CP072662.1	BW25113	Lab strain
NZ_CP050289.1	pK19EC149	Clinical isolate	NZ_CP072663.1	BW25113	Lab strain
NZ_CP053736.1	CP8-3_Sichuan	Clinical isolate	NZ_CP034658.1	ATCC	Lab strain
NZ_CP058571.1	EC2	Clinical isolate	NZ_CP070962.1	K-12	Lab strain
NZ_AP021946.1	WP2-S18-ESBL-08	Clinical isolate	NZ_CP077969.2	JM83	Lab strain
NZ_AP022030.1	WP3-S18-ESBL-07	Clinical isolate	NZ_CP080399.1	DH5	Lab strain
NZ_AP022105.1	WP5-S17-ESBL-10	Clinical isolate	NZ_CP080620.1	TOP10	Lab strain
NZ_AP022177.1	WP7-S17-ESBL-04	Clinical isolate	NZ_CP081008.1	XL10-gold-ultracompetent	Lab strain
NZ_CP047277.1	N18-04097	Clinical isolate	NZ_CP081007.1	XL1-Blue	Lab strain
NZ_CP050197.1	RH-004-MS	Clinical isolate	NZ_CP081009.1	Stbl4	Lab strain
NZ_CP050200.1	RH-024-MS	Clinical isolate	NZ_CP076404.1	K-12	Lab strain
NZ_CP050203.1	RH-045-MS	Clinical isolate	NZ_CP084529.1	Origami2-DE3	Lab strain
NZ_CP060748.1	EC96	Clinical isolate	NZ_CP084899.2	W3110-P	Lab strain
NZ_CP062815.1	Res13-Sevr-PER06-05-b-C	Clinical isolate	NZ_CP076470.1	DH5a	Lab strain
NZ_CP062826.1	Res13-Lact-PER12-33-A	Clinical isolate	NZ_CP093221.1	ER2683	Lab strain
NZ_CP062832.1	Res13-Lact-PER04-33	Clinical isolate	NZ_CP087136.1	JW5503	Lab strain
NZ_CP062838.1	Res13-Lact-PER02-33	Clinical isolate	NZ_CP097881.1	K-12 sub	Lab strain
NZ_CP062883.1	Res13-Lact-PEA06-10	Clinical isolate	NZ_CP097882.1	K-12 sub	Lab strain
NZ_CP062863.1	Res13-Lact-PEB14-36	Clinical isolate	NZ_CP097883.1	K-12 sub	Lab strain
NZ_CP062895.1	Res13-Lact-ER02-39	Clinical isolate	NZ_CP097884.1	K-12 sub	Lab strain
NZ_CP062908.1	Res13-Lact-EB12-05	Clinical isolate	NZ_CP110999.1	J53-p133	Lab strain
NZ_CP065619.1	FDAARGOS_942	Clinical isolate	NZ_CP111004.1	J53-p131	Lab strain
NZ_AP023286.1	2018-18.25PC	Clinical isolate	NZ_CP111008.1	J53-p130	Lab strain
NZ_CP067994.1	CV601	Clinical isolate	NZ_CP117962.1	JM109	Lab strain
NZ_CP069459.1	FDAARGOS_1249	Clinical isolate	NZ_CP119400.1	MRE162 sub	Lab strain
NZ_CP069542.1	FDAARGOS_1248	Clinical isolate	NZ_CP119402.1	MRE162 sub	Lab strain
NZ_CP070026.1	FDAARGOS_1337	Clinical isolate	NZ_CP119404.1	MRE162 sub	Lab strain
NZ_CP063473.1	EM18-18-42	Clinical isolate	NZ_CP110018.1	DH10B	Lab strain
NZ_CP063483.1	EM03-18-08	Clinical isolate	NZ_CP122319.1	BW25113	Lab strain
NZ_CP063487.1	EF7-18-51	Clinical isolate	NZ_CP128218.1	TG1	Lab strain
NZ_CP063499.1	EH10-18-47	Clinical isolate	NZ_CP132550.1	XL1-Blue	Lab strain
NZ_CP063503.1	EH09-18-41	Clinical isolate	NZ_CP132594.1	K-12	Lab strain
NZ_CP066366.1	AR	Clinical isolate	NZ_CP125695.1	DH5-alpha-pBACpAK-KAN	Lab strain
NZ_CP067250.1	ESBL_DR28	Clinical isolate	NZ_CP125701.1	DH5-alpha- pBACpAK-COL	Lab strain
NZ_CP074120.1	EC16	Clinical isolate	NZ_CP125702.1	DH5-alpha-pD25466-pBACpAK-KAN-FM3-C3-200-2	Lab strain
NZ_CP059119.1	Ec42	Clinical isolate	NZ_CP125706.1	DH5-alpha-pD25466-pBACpAK-COL-FM1-C1-10-1-5	Lab strain
NZ_CP082127.1	NDM_12_14482	Clinical isolate	NZ_CP125710.1	DH5-alpha-pD25466-pBACpAK-COL-FM1-C2-10-1-PPY	Lab strain
NZ_CP082129.1	NDM_11.16372	Clinical isolate	NZ_CP125713.1	DH5-alpha-pD25466-pBACpAK-COL-FM1-C2-10-1-1	Lab strain
NZ_CP083877.1	NDM4	Clinical isolate	NZ_CP125715.1	DH5-alpha-pD25466-pBACpAK-COL-FM3-C3-10-1-6	Lab strain
NZ_CP083872.1	NDM5	Clinical isolate	NZ_CP125676.1	DH5-alpha-pD25466-pBACpAK-COL-FM1-C1-10-1-2	Lab strain

Genome	Strain name	Source	Genome	Strain name	Source
NZ_CP083886.1	NDM2	Clinical isolate	NZ_CP125681.1	DH5-alpha-pD25466-pBACpAK-COL-FM1-C2-10-1-SUB-2	Lab strain
NZ_CP083880.1	NDM3	Clinical isolate	NZ_CP125699.1	DH5-alpha-pD25466-pBACpAK-COL-FM3-C3-10-1-7	Lab strain
NZ_CP083869.1	NDM6	Clinical isolate	NZ_CP125688.1	DH5-alpha-pD25466-pBACpAK-COL-FM3-C3-16hr-CCT-27-7	Lab strain
NZ_CP083870.1	NDM6	Clinical isolate	NZ_CP125679.1	DH5-alpha-pD25466-pBACpAK-COL-FM1-C1-10-1-1	Lab strain
NZ_CP083871.1	NDM6	Clinical isolate	NZ_CP149794.1	EC600	Lab strain
NZ_CP084026.1	035053C1pKPC2 Vir 090515B1	Clinical isolate	NZ_CP152465.1	Sel(0	Lab strain
NZ_AP025209.2	2017.06.04CC	Clinical isolate	NZ_CP152466.1	Sel(eng_pnp-mut)	Lab strain
NZ_CP060987.1	EC31	Clinical isolate	NZ_CP152467.1	Sel(aux	Lab strain
NZ_CP060867.1	EC29	Clinical isolate	NZ_CP152468.1	Sel(hit)	Lab strain
NZ_CP060880.1	EC26	Clinical isolate	NZ_CP152469.1	Sel(aux)	Lab strain
NZ_CP060892.1	EC22	Clinical isolate	NZ_CP154880.1	K-12-Ho	Lab strain
NZ_CP060928.1	EC13	Clinical isolate	NZ_CP155065.1	HB101-Ho	Lab strain
NZ_CP060938.1	EC11	Clinical isolate	NZ_CP144891.1	MG1655p	Lab strain
NZ_CP043279.1	WCHEC025157	Clinical isolate	NZ_CP167195.1	C600	Lab strain
NZ_CP060963.1	EC7	Clinical isolate	NZ_CP169294.1	MG1655-pvir	Lab strain
NZ_CP060974.1	EC4	Clinical isolate	NZ_CP169569.1	MC1061	Lab strain
NZ_CP060979.1	EC3	Clinical isolate	NZ_CP169632.1	L-formLTE	Lab strain
NZ_CP092973.1	WCHEC005215	Clinical isolate	NZ_CP169633.1	L-form0	Lab strain
NZ_CP091756.1	LC-1302-2020	Clinical isolate	NZ_CP169634.1	K-12 sub	Lab strain
NZ_CP093226.1	EC-16-35	Clinical isolate	NZ_CP174267.1	K-12 sub	Lab strain
NZ_CP093283.1	EC-14-2-9	Clinical isolate	NZ_CP178206.1	K-12	Lab strain
NZ_CP093919.1	ST167	Clinical isolate	NZ_CP178859.1	HB101	Lab strain
NZ_CP086500.1	Ec-050-T5-MAC	Clinical isolate	NZ_CP181475.1	C600-p2-V	Lab strain
NZ_CP086490.1	Ec-050-T5-ESBL	Clinical isolate	NZ_CP181477.1	C600-p2-3	Lab strain
NZ_CP086480.1	Ec-050-T20-MAC	Clinical isolate	NZ_CP165600.1	K-12 sub	Lab strain
NZ_CP086470.1	Ec-050-T20-ESBL	Clinical isolate	NZ_CP184671.1	HB101	Lab strain
NZ_CP086510.1	Ec-050-T0	Clinical isolate	NZ_CP184740.1	LTE950	Lab strain
NZ_CP080070.1	19SZHZ713Rt	Clinical isolate	NZ_CP184741.1	LTE800	Lab strain
NZ_CP083610.1	EC2648-Vir1	Clinical isolate	NZ_CP184743.1	LTE0	Lab strain
NZ_CP083990.1	EC2648-Vir2	Clinical isolate	NZ_CP184745.1	K-12	Lab strain
NZ_CP084205.1	EC2648-R1	Clinical isolate	NZ_CP185789.1	M15	Lab strain
NZ_CP084207.1	EC2648-R1-1	Clinical isolate	NZ_CP193717.1	DSM	Lab strain
NZ_CP084209.1	EC2648-R2	Clinical isolate	NZ_CP193896.1	BW25113	Lab strain
NZ_CP116094.1	DETEC-S560	Clinical isolate	NZ_CP107553.1	MG1655_insertion	Lab strain
NZ_CP116159.1	DETEC-P351	Clinical isolate	NZ_CP194060.1	BW25113	Lab strain
NZ_CP116188.1	DETEC-E223	Clinical isolate	NZ_CP150878.1	JM109	Lab strain
NZ_CP116046.1	EC812A1	Clinical isolate	NZ_LR217818.1	ER1821R	Lab strain
NZ_CP116110.1	DETEC-P80	Clinical isolate	NZ_CP174268.1	RiboJ-M2-day21	Lab strain
NZ_CP087564.1	CM2010-0578/1	Clinical isolate	NZ_CP157352.1	MG1655::laclq-mcherry	Lab strain
NZ_CP122880.1	ETEC1716	Clinical isolate	NZ_CP019629.1	MGY	Lab strain
NZ_CP122895.1	ETEC1707F	Clinical isolate	NZ_CP013952.1	HST04	Lab strain
NZ_CP122904.1	ETEC1706	Clinical isolate	NZ_CP022466.1	DS1	Lab strain
NZ_CP122929.1	ETEC1702	Clinical isolate	NZ_CP026026.1	CAR	Lab strain
NZ_CP122620.1	ETEC4090	Clinical isolate	NZ_CP026027.1	LIM	Lab strain
NZ_CP122625.1	ETEC4089	Clinical isolate	NZ_CP026028.1	CIT	Lab strain
NZ_CP122660.1	ETEC4073	Clinical isolate	NZ_CP026346.1	7FA	Lab strain

Genome	Strain name	Source	Genome	Strain name	Source
NZ_CP122758.1	ETEC1739	Clinical isolate	NZ_CP026348.1	6FA	Lab strain
NZ_CP122844.1	ETEC1722	Clinical isolate	NZ_CP026349.1	6A	Lab strain
NZ_CP122859.1	ETEC1720	Clinical isolate	NZ_CP026354.1	3FA	Lab strain
NZ_CP126297.1	IVRI_FBI_592	Clinical isolate	NZ_CP026355.1	3A	Lab strain
NZ_CP128951.1	TUM2805	Clinical isolate	NZ_CP026359.1	2_0	Lab strain
NZ_CP115361.1	CUVET20-1667	Clinical isolate	NZ_CP026343.1	9A	Lab strain
NZ_CP115370.1	CUVET17-972	Clinical isolate	NZ_CP026342.1	9FA	Lab strain
NZ_CP133453.1	CREC8	Clinical isolate	NZ_CP026345.1	8A	Lab strain
NZ_CP134370.1	TUM12357	Clinical isolate	NZ_CP026351.1	5A	Lab strain
NZ_CP135680.1	TUM3755	Clinical isolate	NZ_CP026356.1	2FA	Lab strain
NZ_CP140504.1	2023CK-01341	Clinical isolate	NZ_CP026360.1	1FA	Lab strain
NZ_CP138910.1	EDEC1	Clinical isolate	NZ_CP026344.1	8FA	Lab strain
NZ_CP141581.1	ECO-104	Clinical isolate	NZ_CP026347.1	7A	Lab strain
NZ_CP143627.1	FJ_Ec_21	Clinical isolate	NZ_CP026350.1	5FA	Lab strain
NZ_AP027470.1	15.03CC	Clinical isolate	NZ_CP026352.1	4FA	Lab strain
NZ_AP027716.1	TBU0218_10-1CC	Clinical isolate	NZ_CP026353.1	4A	Lab strain
NZ_AP027748.1	2017.02.03CC	Clinical isolate	NZ_CP026357.1	2F_0	Lab strain
NZ_AP027759.1	2017-02-1CC	Clinical isolate	NZ_CP026358.1	2A	Lab strain
NZ_AP027769.1	2017.03.02CC	Clinical isolate	NZ_CP026361.1	1A	Lab strain
NZ_AP027822.1	2017.17.01CC	Clinical isolate	NZ_CP028703.1	ME8067	Lab strain
NZ_AP027829.1	2017.17.03CC	Clinical isolate	NZ_CP034953.2	MT102	Lab strain
NZ_AP027865.1	2017.20.01CC	Clinical isolate	NZ_CP037857.2	AM187	Lab strain
NZ_AP027889.1	2018-06-4CC	Clinical isolate	NZ_CP029240.1	BH214	Lab strain
NZ_AP027902.1	2018-11-1BCC	Clinical isolate	NZ_CP029238.1	CJ236	Lab strain
NZ_CP144926.1	SCPM-O-B-7615	Clinical isolate	NZ_CP029239.1	BH212	Lab strain
NZ_CP144928.1	SCPM-O-B-12142	Clinical isolate	NZ_CP040643.1	BE104	Lab strain
NZ_CP148275.1	Z1323PEC0012	Clinical isolate	NZ_CP042843.1	JME67	Lab strain
NZ_CP148385.1	Z1323HEC0011	Clinical isolate	NZ_CP042845.1	JME65	Lab strain
NZ_CP148405.1	Z1323CEC0018	Clinical isolate	NZ_CP042846.1	JME64	Lab strain
NZ_CP148409.1	Z1323CEC0017	Clinical isolate	NZ_CP042844.1	JME66	Lab strain
NZ_CP148416.1	Z1323CEC0016	Clinical isolate	NZ_CP062239.1	AML001_ev03	Lab strain
NZ_CP148426.1	Z1323CEC0014	Clinical isolate	NZ_CP062240.1	AML001_ev02	Lab strain
NZ_CP148427.1	Z1323CEC0013	Clinical isolate	NZ_CP062241.1	AML001_ev01	Lab strain
NZ_CP148435.1	Z1323CEC0012	Clinical isolate	NZ_CP062242.1	AML001_par	Lab strain
NZ_CP148440.1	Z1323CEC0011	Clinical isolate	NZ_CP062243.1	AML003_ev03	Lab strain
NZ_CP148445.1	Z1323CEC0010	Clinical isolate	NZ_CP062244.1	AML003_ev02	Lab strain
NZ_CP148453.1	Z1323CEC0009	Clinical isolate	NZ_CP062245.1	AML003_ev01	Lab strain
NZ_CP148461.1	Z1323CEC0008	Clinical isolate	NZ_CP062247.1	AML002_ev03	Lab strain
NZ_CP148463.1	Z1323CEC0007	Clinical isolate	NZ_CP062246.1	AML003_par	Lab strain
NZ_CP148466.1	Z1323CEC0006	Clinical isolate	NZ_CP062248.1	AML002_ev02	Lab strain
NZ_CP148493.1	Z1323CEC0003	Clinical isolate	NZ_CP062249.1	AML002_ev01	Lab strain
NZ_CP148507.1	Z1323CEC0001	Clinical isolate	NZ_CP062250.1	AML002_par	Lab strain
NZ_CP150989.1	CUVET16-819	Clinical isolate	NZ_CP064678.1	CETR3G40	Lab strain
NZ_CP157243.1	2024CK-00535	Clinical isolate	NZ_CP064679.1	CETR2G40	Lab strain
NZ_CP166435.1	2024CK-00616	Clinical isolate	NZ_CP064680.1	CETR1G40	Lab strain
NZ_CP157325.2	J53-KPC_Vir-TC	Clinical isolate	NZ_CP064681.1	BZKR3G40	Lab strain
NZ_CP158187.1	EC0900	Clinical isolate	NZ_CP064682.1	BZKR2G40	Lab strain
NZ_CP158674.1	J53-KPC_Vir-P15	Clinical isolate	NZ_CP064683.1	BZKR1G40	Lab strain
NZ_CP166186.1	CRE78	Clinical isolate	NZ_CP072054.1	K1508	Lab strain
NZ_CP100890.1	ET881	Clinical isolate	NZ_CP068279.1	CY708	Lab strain
NZ_CP100922.1	ET734	Clinical isolate	NZ_CP068281.1	CY706	Lab strain
NZ_CP100933.1	ET665	Clinical isolate	NZ_CP068283.1	CY598	Lab strain
NZ_CP100941.1	ET625	Clinical isolate	NZ_CP084893.2	g94	Lab strain
NZ_CP166759.1	CRE389	Clinical isolate	NZ_CP084894.2	g83	Lab strain
NZ_CP168035.1	EC23HZZ201	Clinical isolate	NZ_CP084895.2	g74	Lab strain

Genome	Strain name	Source	Genome	Strain name	Source
NZ_CP151643.1	Ec73552	Clinical isolate	NZ_CP084898.2	gw25	Lab strain
NZ_CP140466.1	GZ04-0083	Clinical isolate	NZ_CP084896.2	g62	Lab strain
NZ_CP148498.1	Z1323CEC0002	Clinical isolate	NZ_CP084897.2	gb37	Lab strain
NZ_CP148483.1	Z1323CEC0004	Clinical isolate	NZ_CP091065.1	DOC14	Lab strain
NZ_CP148474.1	Z1323CEC0005	Clinical isolate	NZ_CP054665.1	IGC_EcoliInv_1.1	Lab strain
NZ_CP150075.1	ECSD228	Clinical isolate	NZ_CP079992.1	KMS2001	Lab strain
NZ_CP171883.1	STECUZB006	Clinical isolate	NZ_CP079993.1	EV36	Lab strain
NZ_CP125918.1	IVRI_FBI_601	Clinical isolate	NZ_CP079991.1	KMS2005	Lab strain
NZ_CP125919.1	IVRI_FBI_602	Clinical isolate	NZ_CP093368.1	EV36	Lab strain
NZ_CP182912.1	FAHZZU6014	Clinical isolate	NZ_CP093369.1	EV36	Lab strain
NZ_CP188128.1	TUM22153	Clinical isolate	NZ_CP101915.1	Evo1	Lab strain
NZ_CP188129.1	TUM22199	Clinical isolate	NZ_CP104206.1	hubt-sl	Lab strain
NZ_CP188130.1	TUM22762	Clinical isolate	NZ_CP104326.1	THB42-F1	Lab strain
NZ_CP188132.1	TUM22763	Clinical isolate	NZ_CP104328.1	THB42-F2	Lab strain
NZ_CP188134.1	TUM22765	Clinical isolate	NZ_CP104330.1	THB42-F3	Lab strain
NZ_CP188136.1	TUM22766	Clinical isolate	NZ_CP104442.1	HBUT-L	Lab strain
NZ_CP188138.1	TUM22769	Clinical isolate	NZ_CP102378.1	JB41	Lab strain
NZ_CP188141.1	TUM23331	Clinical isolate	NZ_CP102379.1	BM28	Lab strain
NZ_CP188143.1	TUM23332	Clinical isolate	NZ_CP102380.1	BM28	Lab strain
NZ_CP188147.1	TUM23337	Clinical isolate	NZ_CP102702.1	9A-1-1	Lab strain
NZ_CP188145.1	TUM23335	Clinical isolate	NZ_CP102705.1	9A-2-7	Lab strain
NZ_CP188149.1	TUM23339	Clinical isolate	NZ_CP102708.1	9A-3-9	Lab strain
NZ_CP188151.1	TUM23341	Clinical isolate	NZ_CP102711.1	9A-4-9	Lab strain
NZ_CP188153.1	TUM24562	Clinical isolate	NZ_CP110859.1	TSW4848-W1	Lab strain
NZ_CP188155.1	TUM24563	Clinical isolate	NZ_CP110014.1	HE_100-10	Lab strain
NZ_CP188157.1	TUM24564	Clinical isolate	NZ_CP110016.1	HE_100	Lab strain
NZ_CP188159.1	TUM24565	Clinical isolate	NZ_CP110017.1	HE_50	Lab strain
NZ_CP188161.1	TUM24566	Clinical isolate	NZ_CP122317.1	HT-948	Lab strain
NZ_CP188163.1	TUM24568	Clinical isolate	NZ_CP122315.1	HT-947	Lab strain
NZ_CP188165.1	TUM24569	Clinical isolate	NZ_CP122316.1	HT-946	Lab strain
NZ_CP188167.1	TUM24570	Clinical isolate	NZ_CP122318.1	HT873X1	Lab strain
NZ_CP188169.1	TUM24769	Clinical isolate	NZ_CP136397.1	DCM272-43-68	Lab strain
NZ_CP190036.1	EC17CM12	Clinical isolate	NZ_CP136398.1	DCM200-113	Lab strain
NZ_CP190333.1	EC17CM13	Clinical isolate	NZ_CP136396.1	DCM188-6	Lab strain
NZ_CP191804.1	EcE.CRE.COL	Clinical isolate	NZ_CP107281.1	JCL301	Lab strain
NZ_CP192729.1	EC7	Clinical isolate	NZ_CP137705.1	FELIX_MS724	Lab strain
NZ_CP192525.1	TUM22760	Clinical isolate	NZ_CP137709.1	FELIX_MS771	Lab strain
NZ_CP192739.1	TUM22761	Clinical isolate	NZ_CP137718.1	FELIX_MS772	Lab strain
NZ_CP192741.1	TUM22764	Clinical isolate	NZ_CP148116.1	FELIX_MS508	Lab strain
NZ_CP192743.1	TUM22767	Clinical isolate	NZ_CP148119.1	FELIX_MS51	Lab strain
NZ_CP192527.1	TUM22768	Clinical isolate	NZ_CP148127.1	FELIX_MS59	Lab strain
NZ_CP192745.1	TUM22775	Clinical isolate	NZ_CP148132.1	FELIX_MS75	Lab strain
NZ_CP192529.1	TUM23349	Clinical isolate	NZ_CP148133.1	FELIX_MS78	Lab strain
NZ_CP192747.1	TUM24567	Clinical isolate	NZ_CP157088.1	NO_STRAIN_FOUND	Lab strain
NZ_CP194907.1	FAHZZU7007	Clinical isolate	NZ_CP163516.1	FELIX_MS516	Lab strain
NZ_CP194957.1	EC15	Clinical isolate	NZ_AP036058.1	FS1576	Lab strain
NZ_CP194969.1	EC28	Clinical isolate	NZ_CP174507.1	Trp01	Lab strain
NZ_CP195093.1	TUM1509	Clinical isolate	NZ_CP129523.1	YJX	Lab strain
NZ_LT601384.1	MS 143-1	Clinical isolate	NZ_CP008801.1	KLY	Environmental
NZ_LT615372.1	HUSEC2011	Clinical isolate	NZ_CP011124.1	USML2	Environmental
NZ_LT615378.1	MS 116-1	Clinical isolate	NZ_CP010143.1	D4	Environmental
NZ_LT615375.1	MS 175-1	Clinical isolate	NZ_CP010152.1	D9	Environmental
NZ_LT615373.1	MS 146-1	Clinical isolate	NZ_CP010172.1	H8	Environmental
NZ_LT615374.1	MS 196-1	Clinical isolate	NZ_CP010231.1	S30	Environmental
NZ_LT615376.1	MS 143-1	Clinical isolate	NZ_CP019778.1	NCTC86	Environmental

Genome	Strain name	Source	Genome	Strain name	Source
NZ_LT615377.1	MS 143-1	Clinical isolate	NZ_CP019005.1	Ecol_AZ155	Environmental
NZ_LT615379.1	MS 143-1	Clinical isolate	NZ_CP022414.1	AT01	Environmental
NZ_LT615371.1	MS 116-1	Clinical isolate	NZ_CP023383.1	1223	Environmental
NZ_LT838196.1	MS 196-1	Clinical isolate	NZ_CP023371.1	1283	Environmental
NZ_LT838200.1	MS 146-1	Clinical isolate	NZ_CP025048.1	SC516	Environmental
NZ_LR134082.1	MS 143-1	Clinical isolate	NZ_CP026612.1	DTU-1	Environmental
NZ_LR134152.1	MS 116-1	Clinical isolate	NZ_CP027105.1	RM14721	Environmental
NZ_LR134216.1	MS 175-1	Clinical isolate	NZ_CP027701.1	675SK2	Environmental
NZ_LR134231.1	MS 146-1	Clinical isolate	NZ_CP027118.1	26561	Environmental
NZ_LR134239.1	MS 196-1	Clinical isolate	NZ_CP029973.1	51008369SK1	Environmental
NZ_LR134295.1	MS 143-1	Clinical isolate	NZ_CP032992.1	W5-6	Environmental
NZ_LR134296.1	MS 116-1	Clinical isolate	NZ_CP034589.1	L37	Environmental
NZ_LR881938.1	SQ88	Clinical isolate	NZ_CP034745.1	L100	Environmental
NZ_LR890603.1	SQ2203	Clinical isolate	NZ_CP034727.1	L41-1	Environmental
NZ_LR890606.1	456_ECOL 2310_5241_450074	Clinical isolate	NZ_CP034734.1	L53	Environmental
NZ_LR898868.1	SQ88	Clinical isolate	NZ_CP039298.1	PigCaeca_1	Environmental
NZ_CP007594.1	SEC470	Clinical isolate	NZ_CP040664.1	KR2009	Environmental
NZ_CP011061.1	Sanji	Clinical isolate	NZ_CP040663.1	EK2009	Environmental
NZ_CP060709.1	ST18	Clinical isolate	NZ_CP042470.1	A50	Environmental
NZ_CP083410.1	RP437	Clinical isolate	NZ_CP042350.1	RM10410	Environmental
NZ_AGTD01000001.1	UMNF18	Clinical isolate	NZ_CP044410.1	ecMN1F	Environmental
NZ_CP010371.1	6409	Clinical isolate	NZ_CP047594.1	LD27-1	Environmental
NZ_CP011134.1	VR50	Clinical isolate	NZ_CP048310.1	32-4	Environmental
NZ_CP013483.1	Y5	Clinical isolate	NZ_CP048330.1	10	Environmental
NZ_OZ121069.1	364_BP0073	Clinical isolate	NZ_CP048371.1	163	Environmental
NZ_CP017631.1	SLK172	Clinical isolate	NZ_CP048367.1	164	Environmental
NZ_CP017980.1	CH611_eco	Clinical isolate	NZ_CP049086.1	pV01-15-R02-025-053	Environmental
NZ_CP086340.1	ESY003	Clinical isolate	NZ_CP033158.1	CM	Environmental
NZ_CP086336.1	EBJ001	Clinical isolate	NZ_CP049299.1	pV01-18-E02-025-051	Environmental
NZ_CP086342.1	ESY001	Clinical isolate	NZ_CP049353.1	T28R	Environmental
NZ_CP086338.1	EJN001	Clinical isolate	NZ_CP053787.1	J31	Environmental
NZ_CP086556.1	ESY002	Clinical isolate	NZ_CP055256.1	AH25	Environmental
NZ_CP020514.1	219	Clinical isolate	NZ_CP050044.1	74-2	Environmental
NZ_CP029180.1	H9Ecoli	Clinical isolate	NZ_CP050040.1	63-2	Environmental
NZ_CP029574.1	DA33133	Clinical isolate	NZ_CP050036.1	58-3	Environmental
NZ_CP029492.1	HS30-1	Clinical isolate	NZ_CP050031.1	33-6	Environmental
NZ_CP023749.1	RTdelA_B_UU3	Clinical isolate	NZ_CP059723.1	BUCT2930	Environmental
NZ_CP023834.1	4/2-1	Clinical isolate	NZ_CP059931.1	28.1	Environmental
NZ_CP025903.1	300709	Clinical isolate	NZ_CP069712.1	EA13	Environmental
NZ_CP035350.1	BR32-DEC	Clinical isolate	NZ_CP070232.1	10R	Environmental
NZ_CP048439.1	NBRC	Clinical isolate	NZ_CP070280.1	S2	Environmental
NZ_CP049081.1	p10A	Clinical isolate	NZ_CP046025.1	C439	Environmental
NZ_CP050382.1	52148	Clinical isolate	NZ_CP046416.1	LZYWN_4	Environmental
NZ_CP051716.1	SCU-118	Clinical isolate	NZ_CP071521.1	11202	Environmental
NZ_CP053284.1	SCU-104	Clinical isolate	NZ_CP063979.1	P1	Environmental
NZ_AP023219.2	M505	Clinical isolate	NZ_CP063956.1	P3	Environmental
NZ_AP023226.1	YJ3	Clinical isolate	NZ_CP063969.1	P8	Environmental
NZ_AP023230.2	YJ4	Clinical isolate	NZ_CP063958.1	P68	Environmental
NZ_AP023235.1	YJ6	Clinical isolate	NZ_CP067299.1	ABW_A33	Environmental
NZ_CP058948.1	SY3626C5	Clinical isolate	NZ_CP067303.1	ABW_A32	Environmental
NZ_CP059043.1	SY3626C1	Clinical isolate	NZ_CP053211.1	QDFD216	Environmental
NZ_CP058661.1	5008	Clinical isolate	NZ_CP072986.1	T159A	Environmental
NZ_CP059288.1	1506	Clinical isolate	NZ_CP076646.1	GD33	Environmental
NZ_CP060057.1	G8	Clinical isolate	NZ_CP069386.1	XH1815	Environmental

Genome	Strain name	Source	Genome	Strain name	Source
NZ_CP060061.1	G6/7	Clinical isolate	NZ_CP067399.1	XJW9B263	Environmental
NZ_CP060083.1	G1/2	Clinical isolate	NZ_CP068041.1	XJW9B277	Environmental
NZ_AP023427.1	HUE1	Clinical isolate	NZ_CP080126.1	PK8568	Environmental
NZ_CP062970.1	188B	Clinical isolate	NZ_CP080139.1	PK8241	Environmental
NZ_CP062967.1	188	Clinical isolate	NZ_CP080253.1	PC6	Environmental
NZ_CP058342.1	W60	Clinical isolate	NZ_CP080223.1	P13-6	Environmental
NZ_CP048821.1	QEC11-421	Clinical isolate	NZ_CP087110.1	PJ-T13	Environmental
NZ_CP063332.1	BS74R-D	Clinical isolate	NZ_CP053855.1	258E	Environmental
NZ_CP070902.1	99COLEC	Clinical isolate	NZ_CP058719.1	M159-1	Environmental
NZ_CP069657.1	MIN12	Clinical isolate	NZ_CP091427.1	1000C-3	Environmental
NZ_CP069666.1	MIN11	Clinical isolate	NZ_CP028760.1	A14	Environmental
NZ_CP069682.1	MIN9	Clinical isolate	NZ_CP091652.1	KMi025	Environmental
NZ_CP062924.1	179	Clinical isolate	NZ_CP091662.1	KFu023	Environmental
NZ_CP062927.1	179A	Clinical isolate	NZ_CP091691.1	KMi006	Environmental
NZ_CP062933.1	71	Clinical isolate	NZ_AP025180.1	JP538	Environmental
NZ_CP062930.1	91A	Clinical isolate	NZ_CP093068.1	BR1220	Environmental
NZ_CP047010.1	19-5	Clinical isolate	NZ_CP098739.1	KU_Poultry_13_Ecoli	Environmental
NZ_CP072858.1	184/2aE	Clinical isolate	NZ_CP100525.1	LH13-b	Environmental
NZ_CP104384.1	JNQH498	Clinical isolate	NZ_CP101666.1	M171-1	Environmental
NZ_CP091925.1	JNQH497	Clinical isolate	NZ_CP075737.1	CPF6	Environmental
NZ_CP076318.1	Survcare045	Clinical isolate	NZ_CP075722.1	CPE35	Environmental
NZ_CP035822.1	WS4437A-1	Clinical isolate	NZ_CP073929.1	MB64	Environmental
NZ_CP035865.1	E275	Clinical isolate	NZ_CP073932.1	MB57	Environmental
NZ_CP079735.1	GB2005	Clinical isolate	NZ_CP073949.1	MB50	Environmental
NZ_CP079736.1	GB2005-delet-a-f	Clinical isolate	NZ_CP073962.1	MB31	Environmental
NZ_CP081711.1	AH21	Clinical isolate	NZ_CP073974.1	MB22	Environmental
NZ_CP085060.1	P30_L1_05.20	Clinical isolate	NZ_CP073981.1	MB19	Environmental
NZ_CP085074.1	P21_SE1_04.20	Clinical isolate	NZ_CP073988.1	MB10	Environmental
NZ_CP091155.1	NEC1	Clinical isolate	NZ_CP073996.1	PM7	Environmental
NZ_CP091162.1	NEC10	Clinical isolate	NZ_CP074001.1	PM4	Environmental
NZ_CP091164.1	NEC12	Clinical isolate	NZ_CP074007.1	PD4	Environmental
NZ_CP092702.1	S-P-C-029.01	Clinical isolate	NZ_CP074024.1	PK5	Environmental
NZ_CP028732.1	H3	Clinical isolate	NZ_CP074047.1	PD7	Environmental
NZ_CP095125.1	Iso00270	Clinical isolate	NZ_CP097213.1	BE311	Environmental
NZ_CP095155.1	Iso00041	Clinical isolate	NZ_CP103972.1	DLI.5a	Environmental
NZ_AP023433.1	M719	Clinical isolate	NZ_CP103974.1	DLI.6n	Environmental
NZ_CP097426.1	C289	Clinical isolate	NZ_CP079767.1	SF10	Environmental
NZ_CP097430.1	C288	Clinical isolate	NZ_CP079770.1	HL15	Environmental
NZ_CP099588.1	EMG2	Clinical isolate	NZ_CP104114.1	USDA-ARS-USMARC-51408	Environmental
NZ_CP099590.1	WG1	Clinical isolate	NZ_CP104509.1	E2	Environmental
NZ_CP117001.1	MLI108K2	Clinical isolate	NZ_CP104472.1	UC4224	Environmental
NZ_CP117049.1	MLI106K2	Clinical isolate	NZ_CP104788.1	SHP24	Environmental
NZ_CP117053.1	MLI106K4	Clinical isolate	NZ_CP123255.1	YZMc17-1	Environmental
NZ_CP117010.1	MLI110	Clinical isolate	NZ_CP082327.1	B2	Environmental
NZ_CP116929.1	MLI105	Clinical isolate	NZ_CP106929.1	MFDS1001074	Environmental
NZ_CP116994.1	MLI108K1	Clinical isolate	NZ_CP109601.1	TL-14	Environmental
NZ_CP103570.1	3045	Clinical isolate	NZ_CP110814.1	THB42-F4	Environmental
NZ_CP103596.1	4848	Clinical isolate	NZ_CP110816.1	THB42-F5	Environmental
NZ_CP103529.1	5270	Clinical isolate	NZ_CP114893.1	CM13	Environmental
NZ_CP103540.1	5264	Clinical isolate	NZ_CP114892.1	CM5	Environmental
NZ_CP103515.1	5444	Clinical isolate	NZ_CP114891.1	CM2	Environmental
NZ_CP103745.1	3090	Clinical isolate	NZ_CP074359.1	XJ6.2	Environmental
NZ_CP103645.1	961	Clinical isolate	NZ_CP114894.1	CM19	Environmental
NZ_CP103758.1	p10B	Clinical isolate	NZ_CP114895.1	CM56	Environmental
NZ_CP104721.1	ECO3183	Clinical isolate	NZ_CP114896.1	CM93	Environmental

Genome	Strain name	Source	Genome	Strain name	Source
NZ_CP117959.1	B-2207	Clinical isolate	NZ_CP047469.1	DY612	Environmental
NZ_CP117971.1	B-1109	Clinical isolate	NZ_CP049110.1	WF5-5-T1	Environmental
NZ_CP120568.1	582	Clinical isolate	NZ_CP049112.1	WF5-5-T2	Environmental
NZ_CP123237.1	4588	Clinical isolate	NZ_CP123272.1	4A_CSF1O9	Environmental
NZ_CP125731.1	GYB01	Clinical isolate	NZ_CP123951.1	TL012	Environmental
NZ_AP025546.1	LR-06	Clinical isolate	NZ_CP110117.1	2-331	Environmental
NZ_AP025551.1	LR-50	Clinical isolate	NZ_AP027946.1	VE-F10	Environmental
NZ_CP128440.1	32	Clinical isolate	NZ_AP027953.1	VE-F5	Environmental
NZ_CP128609.1	HZ13	Clinical isolate	NZ_CP122496.1	a7	Environmental
NZ_CP128790.1	WL7562	Clinical isolate	NZ_CP122507.1	W444	Environmental
NZ_CP082100.1	MS15047	Clinical isolate	NZ_AP027404.1	EC416	Environmental
NZ_CP130447.1	11899A_LBHALD	Clinical isolate	NZ_CP106994.1	SY22A6	Environmental
NZ_CP133854.1	ES4	Clinical isolate	NZ_CP106995.1	SY22B3	Environmental
NZ_CP135502.1	isolateA	Clinical isolate	NZ_CP106996.1	SY22D8	Environmental
NZ_CP132223.1	Jtgc-02-2-Tmex	Clinical isolate	NZ_CP106997.1	SY22B6	Environmental
NZ_CP138407.1	50EVAW	Clinical isolate	NZ_CP106998.1	SY221	Environmental
NZ_CP138423.1	30EVA1	Clinical isolate	NZ_CP106999.1	SY22A4	Environmental
NZ_CP138416.1	39EVA	Clinical isolate	NZ_CP107000.1	SY22B2	Environmental
NZ_CP139657.1	TP10-3	Clinical isolate	NZ_CP107001.1	SY22B5	Environmental
NZ_CP139661.1	TP10-2	Clinical isolate	NZ_CP107002.1	SY22C3	Environmental
NZ_CP139665.1	TP10-1	Clinical isolate	NZ_CP107003.1	SY22B1	Environmental
NZ_CP139669.1	TP6-3	Clinical isolate	NZ_CP107004.1	SY22A3	Environmental
NZ_CP139673.1	TP6-2	Clinical isolate	NZ_CP107005.1	SY22A1	Environmental
NZ_CP139677.1	TP6-1	Clinical isolate	NZ_CP128584.1	VG48B2	Environmental
NZ_CP141075.1	KE58	Clinical isolate	NZ_CP119008.1	CE0011b	Environmental
NZ_AP027474.1	LR-03	Clinical isolate	NZ_CP130706.1	DDG220	Environmental
NZ_AP027494.1	LR-24	Clinical isolate	NZ_CP130710.1	DDG206	Environmental
NZ_AP027515.1	LR-29-1	Clinical isolate	NZ_CP134644.1	918:C	Environmental
NZ_AP027523.1	LR-33	Clinical isolate	NZ_CP136025.1	GDSC2BY4G	Environmental
NZ_AP027543.1	LR-48	Clinical isolate	NZ_CP136993.2	HH35	Environmental
NZ_AP027553.1	LR-58-1	Clinical isolate	NZ_CP139337.1	YZ22MPE54	Environmental
NZ_AP027595.1	LR-64-2	Clinical isolate	NZ_CP139904.1	THN257	Environmental
NZ_AP027599.1	LR-71-1	Clinical isolate	NZ_CP141787.1	K194T1-1018	Environmental
NZ_AP027612.1	LR-72	Clinical isolate	NZ_CP141789.1	K194T1-1017	Environmental
NZ_AP027619.1	PSJ-07	Clinical isolate	NZ_CP142044.1	L9	Environmental
NZ_AP027626.1	PSJ-14	Clinical isolate	NZ_CP142915.1	B19374	Environmental
NZ_AP027632.1	PSJ-16	Clinical isolate	NZ_CP142939.1	B20127	Environmental
NZ_AP027642.1	PSJ-32-1	Clinical isolate	NZ_CP147059.1	JKHS016	Environmental
NZ_AP027666.1	PSS-04	Clinical isolate	NZ_CP155555.1	LZHX12	Environmental
NZ_CP144364.1	H106	Clinical isolate	NZ_CP157958.1	800	Environmental
NZ_CP145700.1	S1-IVC-02-A	Clinical isolate	NZ_CP158503.1	E-ge-1	Environmental
NZ_CP146654.1	MS 124-1	Clinical isolate	NZ_CP160468.1	PEC1009	Environmental
NZ_CP146681.1	MS 196-1	Clinical isolate	NZ_CP160643.1	PEC1003	Environmental
NZ_CP149967.1	SLDY13	Clinical isolate	NZ_CP146890.1	2-C14-1	Environmental
NZ_CP159141.1	10034940-2	Clinical isolate	NZ_CP146893.1	C21-2	Environmental
NZ_CP160025.1	1000	Clinical isolate	NZ_CP158140.1	FDN-9	Environmental
NZ_CP163029.1	W170	Clinical isolate	NZ_CP158147.1	FDN-24	Environmental
NZ_CP168269.1	IH5	Clinical isolate	NZ_CP172152.1	GF31	Environmental
NZ_CP170509.1	30088	Clinical isolate	NZ_CP172173.1	GF124.3	Environmental
NZ_CP173466.1	98227	Clinical isolate	NZ_CP172816.1	BS-01	Environmental
NZ_CP173582.1	98195	Clinical isolate	NZ_CP172867.1	35	Environmental
NZ_CP173486.1	98219	Clinical isolate	NZ_CP139125.1	YZ22PE244	Environmental
NZ_CP173509.1	98215	Clinical isolate	NZ_CP142943.1	B20159	Environmental
NZ_CP173524.1	98212	Clinical isolate	NZ_CP162198.1	STK39-3-E	Environmental
NZ_CP176484.1	N921	Clinical isolate	NZ_CP162185.1	STK78-E	Environmental

Genome	Strain name	Source	Genome	Strain name	Source
NZ_CP181463.1	28I	Clinical isolate	NZ_CP142708.1	T28R	Environmental
NZ_CP181464.1	28G	Clinical isolate	NZ_CP142843.1	SX45	Environmental
NZ_CP181466.1	27D	Clinical isolate	NZ_CP142783.1	SX12	Environmental
NZ_CP181465.1	28A	Clinical isolate	NZ_CP176786.1	XJ23PTE1	Environmental
NZ_CP183153.1	572	Clinical isolate	NZ_CP178240.1	JW-5	Environmental
NZ_CP183161.1	605	Clinical isolate	NZ_CP178548.1	WWCOL-256	Environmental
NZ_CP184016.1	1380	Clinical isolate	NZ_CP180624.1	UPMP2439	Environmental
NZ_CP184155.1	C21	Clinical isolate	NZ_CP180625.1	UPMP2388	Environmental
NZ_CP184262.1	H32	Clinical isolate	NZ_CP180690.1	CUPS03	Environmental
NZ_CP186506.1	R18	Clinical isolate	NZ_CP180700.1	CUPS05	Environmental
NZ_CP187522.1	XSLGL-2	Clinical isolate	NZ_CP182159.1	CUWW12	Environmental
NZ_CP069093.1	TOE17	Clinical isolate	NZ_CP182174.1	CUPF06	Environmental
NZ_CP071124.1	PS200	Clinical isolate	NZ_CP182383.1	Br_178_kylling	Environmental
NZ_CP143392.1	HD12480	Clinical isolate	NZ_CP182336.1	A134_1EC	Environmental
NZ_CP176406.1	BA92	Clinical isolate	NZ_CP154887.1	5735	Environmental
NZ_CP166722.1	CEA106S	Clinical isolate	NZ_CP184992.1	CUPS	Environmental
NZ_AP019189.1	M217	Clinical isolate	NZ_CP074754.1	F3_13_1_3	Environmental
NZ_LR890288.1	MSB1_1A	Clinical isolate	NZ_CP154401.1	TBE6	Environmental
NZ_OW967975.1	10	Clinical isolate	NZ_CP154406.1	TST3	Environmental
NZ_OW968124.1	10	Clinical isolate	NZ_CP154412.1	TF5	Environmental
NZ_OW968288.1	10	Clinical isolate	NZ_CP154596.1	SEV4	Environmental
NZ_CP144930.1	SCPM-O-B-9504	Clinical isolate	NZ_CP151548.1	SEV1	Environmental
NC_000913.3	K-12 sub	Lab strain	NZ_LS992166.1	TO6	Environmental
NC_007779.1	K-12 sub	Lab strain	NZ_LS992171.1	TO73	Environmental
NC_010473.1	K-12 sub	Lab strain	NZ_LS992185.1	3426	Environmental
NC_012759.1	K-12 sub	Lab strain	NZ_LR882052.1	2015-01-466	Environmental
NC_017625.1	DH1	Lab strain	NZ_OY754443.1	F829	Environmental
NZ_CM000960.1	K-12 sub	Lab strain	NZ_OY754453.1	F95	Environmental
NC_017660.1	KO11FL	Lab strain	NZ_OZ261674.1	GENOMIC23-001	Environmental
